# Clinical Profile and Complications Experienced by Chronic Kidney Disease Patients undergoing Hemodialysis in a Tertiary Hospital of Nepal: An Observational Study

**DOI:** 10.31729/jnma.8945

**Published:** 2025-04-30

**Authors:** Bimala Kumari Sah, Imran Khan, Ashesh Dhungana

**Affiliations:** 1 Sharda School of Nursing Science and Research, Sharda University, Greater Noida, India; 2 National Academy of Medical Sciences, Bir Hospital, Kathmandu, Nepal

**Keywords:** *chronic kidney disease*, *complications*, *hemodialysis*, *Nepal*

## Abstract

**Introduction:**

Chronic kidney disease is emerging public health problems globally. Hemodialysis is the commonest treatment modality but it causes some complications and fluctuation in parameters. This study aimed to assess the clinical profile and complications experienced by chronic kidney disease patients undergoing hemodialysis.

**Methods:**

This was an observational study conducted from December, 2023 to February, 2024 Patients aged 20 and above, receiving hemodialysis more than three months were included in the study. Descriptive analysis was done.

**Results:**

Among 110 CKD patients undergoing hemodialysis, 62 (56.36%) were 50 years of age or younger, with a mean 48.76±13.365. The study population included 66 (60%) male and mean age of the patients was 48.76±13.36. In this study group 90 (81.82%) had two session of dialysis per week. Mean Pre-HD systolic blood pressure was 150.64±23.95 mmHg and Post-HD was 147.64±24.38 mmHg. In this study the mean hemoglobin was 9.38±1.85 gm/dl, glucose was 110.85±46.62 mg/dl, urea 91.81±37.73 mg/dl and creatinine 7.94±2.68 mg/dl. In the patient population undergoing dialysis oliguria was observed in 106 (96.36%), weakness 98 (89.09%) and insomnia was observed in 87 (79.09%.

**Conclusions:**

In this study, more than half of the patient were in the age group of less than or equal to 50 years. Common complications included reduced urine output, weakness, and insomnia. Hypertension was the most common underlying condition, often occurring with diabetes.

## INTRODUCTION

Chronic kidney disease (CKD) has become a public health concern around the world. The majority of patients in the underdeveloped countries cannot afford the high expense of managing CKD.^1^ Renal replacement therapy of hemodialysis (HD) is popular in the world and Nepal.^2^

CKD prevalence in South Asia includes 10.2% in India,10.6% in Nepal, 17.3% in Bangladesh, and 23.3% in Pakistan.^3^ In Nepal, 8,000 CKD patients are undergoing haemodialysis (HD).^4^ HD is essential in patient longevity but impacts lives in various ways.^5^ HD can cause many physical and mental health complications.^6^ The complications included hypotension, muscle cramps, shivering, exhaustion, headache, nausea, allergic reactions, back pain, and hypoglycemia.^7^

Understanding the clinical profile and complications of CKD patients undergoing hemodialysis is crucial for improving patient care and developing hospital-specific intervention strategies for reducing complications and maintaining health of the hemodialysis patients. By addressing these gaps, this study aimed to identify the clinical profile and complications experienced by CKD patients undergoing hemodialysis in a tertiary hospital of Nepal.

## METHODS

This was an observational cross-section study conducted among 110 CKD patients undergoing hemodialysis at Nephrology department of Bir Hospital, which is a tertiary care hospital located at Kathmandu, Nepal. The study was conducted from December, 2023 to February, 2024 after ethical clearance from the Institutional Review Board (Reference number: 540), Bir-Hospital/NAMS and Nepal Health Research Council (NHRC), Kathmandu (Reference number: 171).

The study population comprised all chronic kidney disease (CKD) patients undergoing hemodialysis at Bir Hospital. The clinical information of all patient was retrieved from the record of hemodialysis unit. The inclusion criteria consisted of patients aged 20 years or older with CKD who had been on maintenance hemodialysis for at least three months and received hemodialysis either twice or thrice a week on an outpatient basis. Patients with dementia, critically ill patients and patients having incomplete record on patient's chart and hemodialysis record sheet were excluded from the study.

A detailed clinical profile, including laboratory findings, was reviewed from patients’ medical charts and hemodialysis records. Experienced complications were assessed through yes or no responses using hemodialysis record sheet. The laboratory findings included Complete Blood Count (CBC), Renal Function Tests (RFTs), serum calcium, serum phosphorus, serum protein, and serum albumin levels. Data analysis was performed using ‘SPSS Statistics for Windows, version 27.0 (SPSS Inc., Chicago, Ill., USA)'where frequency and percentage were reported for binary data, and mean with standard deviation was used for continuous data.

## RESULTS

A record of 110 CKD patient meeting inclusion criteria were analyzed for this study. The study population included 66 (60%) male, the mean age of the patients was 48.76(±13.36) and 64 (58.18%) stayed in rented home. In this study group 90 (81.82%) had two session of dialysis per week and mean duration of dialysis was 45.11±40.87 months. Mean Pre-HD systolic blood pressure was 150.64±23.95 mmHg and Post-HD was 147.64±24.38 mmHg. Similarly, Pre-HD diastolic blood pressure was 80.89±15.78 mmHg and Post-HD was 79.18±12.03mmHg. Body weight of the patient Pre-HD was 58.85±11.69 kg and Post-HD it was 55.80±11.66 kg (Table 1).

**Table 1:** Baseline characteristics of CKD patients treated with hemodialysis (n=110).

Clinical Profile	n (%)
**Age**	
≤50	62 (56.36)
>50	48 (43.63)
**Gender**	
Male	66 (60)
Female	44 (40)
**Residence**	
Own home	46 (41.82)
Rented home	64 (58.18)
**Frequency of Hemodialysis session (week)**	
2 times	90 (81.82)
3 times	20 (18.18)
**Duration of Hemodialysis session (year)**	
<1year	21 (19.09)
1to 3 years	27 (24.55)
3 to 5 years	37 (33.64)
5 to 10 years	15 (13.64)
≥10 years	10 (9.09)
**Pre-HD Systolic Blood Pressure (mm of hg)**	
≤120	8 (7.27)
121 to ≤140	19 (17.27)
> 140	83 (75.45)
**Pre-HD Diastolic Blood Pressure (mm of hg)**	
≤70	15 (13.64)
71 to ≤80	22 (20)
> 80	73 (66.36)
**Post-HD Systolic Blood Pressure (mm of hg)**	
≤120	10 (9.09)
121 to ≤140	19 (17.27)
> 140	81 (73.64)
**Post-HD Diastolic Blood Pressure (mm of hg)**	
≤70	35 (31.82)
71 to ≤80	38 (34.55)
> 80	37 (33.64)
**Pre - HD body weight (kg)**	
30-50	22 (20)
50-70	70 (63.64)
70-90	14 (12.73)
≥90	4 (3.64)
**Post - HD body weight (kg)**	
30-50	46 (41.82)
50-70	50 (45.45)
70-90	11 (10)
≥90	3 (2.73)

In this study the mean hemoglobin was 9.38±1.85 gm/dl, glucose was 110.85±46.62 mg/dl, urea 91.81±37.73 mg/dl and creatinine 7.94±2.68 mg/dl. Similarly mean value for sodium (Na) was 137±3.66 meq/dl, potassium (K) was 4.95±0.85 meq/dl, serum albumin 4.22±0.80 g/dl, calcium (Ca) 9.04±1.29 mg/dl, phosphorus (P) 5.01±1.82 and total protein 7.12±0.92 (Table 2).

**Table 2:** Hematological profile of CKD patients treat­ed with hemodialysis (n=110).

Parameters	n (%)
**Hemoglobin (gm/dL)**	
<6	5 (4.55)
6-10	61 (55.45)
>10	44 (40)
**Glucose (Random, mg/dL)**	
<70	5 (4.55)
70-140	88 (80)
140-200	13 (11.82)
>200	4 (3.64)
**Urea (mg/dL)**	
<50	12 (10.91)
50-150	91 (82.73)
>150	7 (6.36)
**Serum Creatinine (mg/dL)**	
<5	15 (13.64)
5-12	90 (81.82)
>12	5 (4.55)
**Sodium (meq/L)**	
125-135	10 (9.09)
135-150	98 (89.09)
>150	2 (1.82)
**Potassium (meq/L)**	
<3.5	4 (3.64)
3.5-5.5	74 (67.27)
>5.5	32 (29.09)
**Serum Albumin (g/dL)**	
<3.4	11 (10)
3.4-5.5	94 (85.45)
>5.5	5 (4.55)
**Serum Calcium (mg/dL)**	
<8.5	24 (21.82)
8.5-10.5	73 (66.36)
>10.5	13 (11.82)
**Serum Phosphorus (mg/dL)**	
2.5-4.4	47 (42.73)
>4.5	63 (57.27)
**Total Protein (g/dL)**	
<6	11 (10)
6-8	78 (70.91)
>8	21 (19.09)

In the patient population undergoing dialysis oliguria was observed in 106 (96.36%), weakness 98 (89.09%) and insomnia was observed in 87 (79.1%), (Table 3).

**Table 3:** Complications experienced by CKD patients treated with hemodialysis (n=110).

Complications (self-reported by participants)	n (%)
Oliguria	106 (96.36)
Weakness	98 (89.09)
Insomnia	87 (79.09)
Hypertension	86 (78.18)
Feeling of mental distress	72 (65.45)
Anorexia	70 (63.64)
Oedema	68 (61.82)
Headache	66 (60.00)
Restlessness	62 (56.36)
Sweating	62 (56.36)
Muscle Cramps	57 (51.82)
Nausea	43 (39.09)
Hypotension	40 (36.36)
Numbness	34 (30.91)
Fever	34 (30.91)
Vomiting	22 (20.00)
Itching	19 (17.27)
Shortness of breath	18 (16.36)
Convulsion	9 (8.18)
Fistula infection	9 (8.18)
Chest Pain	7 (6.36)

**Figure 1:**
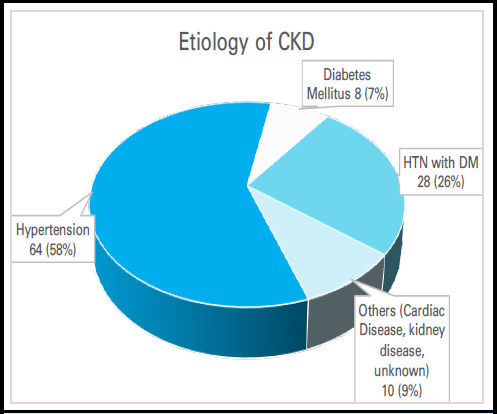
Etiology of chronic kidney disease (n=110).

The most commonly noticed etiology among the CKD patients undergoing hemodialysis was hy[ertension in 64 (58.18%) patient and hypertension with diabetes in 28 (25.45%) of patients (Figure 1).

## DISCUSSION

In this study, among 110 CKD patients with hemodialysis, the range age was 21 to 85 years with mean 48.76±13.36 years, where 62 (56.36%) comprised up to 50 years. This indicates most of the active age group population are suffering from CKD and required HD. This finding is aligned with several other studies done in Nepal.^8,9^ Hemodialysis was administered twice weekly in 90 (81.8%) participants and remaining 20(18.2%) received HD three times a week. In contrast, studies from India and Iran reported 58% and 72.9% of participants received HD three times a week.^10,11^

The most common underlying etiological factor of CKD was hypertension 64 (58.18%) followed by Diabetes Mellitus 8 (7.27%) and both (hypertension with diabetes) 28 (25.45%). These findings are supported by different other studies done in Nepal.^12,13,8^ Furthermore, study from India also showed hypertension is the primary causes of CKD as in 58% of patients, followed by diabetes mellitus in 13%.^10^ But in contrast, some literature shows, many studies from Nepal and outside reported diabetes as the main cause for CKD.^14,15,9^

Regarding Pre-HD Blood pressure, it was reported >140 mm of hg systolic in majority 83 (75.45%) and > 80 mm of hg diastolic in two third 73 (66.36%) of participants with mean 150.64±23.95 and 80.89±15.78 respectively. Almost similar findings of study done in Ethiopia showed mean systolic and diastolic blood pressure 160±21 and 93±13 respectively.^16^

In this study almost two third of the participants 66 (60%) had anemia (Hb < 10gm/dL) with mean 9.38±1.85. Similar other findings are seen as hemoglobin levels were below 10 gm/dl in 90% of the patients.^17^ Similar findings revealed in different other studies in Nepal and outside.^13,15,17-19^ Furthermore, a study carried out in Africa, among 863 patients of ESRD had hemoglobin less than 10 gm/dl. in 90%. ^20^ The presence of erythropoiesis inhibitors or the decrease of erythropoietin production in the kidneys can lead to lower hemoglobin levels. ^20^

Similarly, majority of the participants 95 (86.36%) had hypercreatininemia (>5 mg/dl) with mean 7.94±2.68 d/l. This finding is aligned with study done in Nepal reported Serum creatinine levels showed an upward trend between stages 4 and 5, significantly high level in stage 5.^21^ Other more findings are 32 (29.09%) had hyperkalemia, 24 (21.82%) had hypocalcemia, 63 (57.27%) had hyperphosphatemia. Similar findings were found in study done in Nepal and India.^9,17,18^

The main complications reported by the patients undergoing hemodialysis were oliguria 106 (96.0%) followed by weakness 98 (89.09%), insomnia 87 (79.09%), hypertension 86 (78.18%), feeling of mental distress 72 (65.45%). Similarly, anorexia 70 (63.64%), oedema 68 (61.82%), headache 66 (60%). Furthermore, sweating 62 (56.36%) followed by muscle cramps 57 (51.82%) and hypotension 40 (36.36%), vomiting 22 (20%). Some findings are more and less similar to other studies carried out in Nepal. ^13,17,18^ However, contrast findings are noticed in study done in Brazil are cramps 149 (74.5%), hypotension 141 (70.5%), headache 71 (35.5%), hypertension 48 (24%), vomiting 77 (38.5%).^22^ Similarly, 0.5% of problems were related to drowsiness, mental anguish, sweating, weakness, shortness of breath, numbness, body aches, and tremors.^22^ Most of the literature showed, hypertension is more common complications.^14,17,18^ In spite of using antihypertensive drugs, a lack of dialysis hours, noncompliance with treatment and dietary carelessness may be the reasons for this patient group's poorly managed hypertension. Findings of the study aid in planning for improving patient treatment and care, proper counselling service. Additionally, focus on promoting awareness program on preventive measures and early diagnosis and intervention to the target population. The applicability of findings in different contexts is limited as the study is carried out in single-center.

## CONCLUSIONS

In this study, more than half of the patient were in the age group of less than or equal to 50 years. This study found that most CKD patients on dialysis were middle-aged males, with many living in rented homes. They typically underwent two dialysis sessions per week, which slightly lowered their blood pressure and reduced their weight. Blood tests showed anemia, high urea and creatinine levels, and electrolyte imbalances. Common complications included reduced urine output, weakness, and insomnia. Hypertension was the most common underlying condition, often occurring with diabetes.
